# Jointly Modelling Single Nucleotide Polymorphisms With Longitudinal and Time-to-Event Trait: An Application to Type 2 Diabetes and Fasting Plasma Glucose

**DOI:** 10.3389/fgene.2018.00210

**Published:** 2018-06-14

**Authors:** Mickaël Canouil, Beverley Balkau, Ronan Roussel, Philippe Froguel, Ghislain Rocheleau

**Affiliations:** ^1^Université de Lille, UMR 8199-EGID, Lille, France; ^2^Centre National de la Recherche Scientifique, UMR 8199, Lille, France; ^3^Institut Pasteur de Lille, Lille, France; ^4^Centre for Research in Epidemiology and Population Health, Villejuif, France; ^5^Université Paris-Saclay, Université Paris Sud, UVSQ, UMRS 1018, Villejuif, France; ^6^Institut National de la Santé et de la Recherche Médicale U1018, Centre de Recherche en Épidémiologie et Santé des Populations, Renal and Cardiovascular Epidemiology, UVSQ-UPS, Villejuif, France; ^7^Institut National de la Santé et de la Recherche Médicale U1138 (équipe 2: Pathophysiology and Therapeutics of Vascular and Renal Diseases Related to Diabetes, Centre de Recherches des Cordeliers), Paris, France; ^8^Université Paris 7 Denis Diderot, Sorbonne Paris Cité, Paris, France; ^9^AP-HP, DHU FIRE, Department of Endocrinology, Diabetology, Nutrition, and Metabolic Diseases, Bichat Claude Bernard Hospital, Paris, France; ^10^Department of Genomics of Common Disease, Imperial College London, London, United Kingdom

**Keywords:** joint modelling, survival analysis, longitudinal biomarker, genetic, diabetes, glycaemia

## Abstract

In observational cohorts, longitudinal data are collected with repeated measurements at predetermined time points for many biomarkers, along with other variables measured at baseline. In these cohorts, time until a certain event of interest occurs is reported and very often, a relationship will be observed between some biomarker repeatedly measured over time and that event. Joint models were designed to efficiently estimate statistical parameters describing this relationship by combining a mixed model for the longitudinal biomarker trajectory and a survival model for the time until occurrence of the event, using a set of random effects to account for the relationship between the two types of data. In this paper, we discuss the implementation of joint models in genetic association studies. First, we check model consistency based on different simulation scenarios, by varying sample sizes, minor allele frequencies and number of repeated measurements. Second, using genotypes assayed with the Metabochip DNA arrays (Illumina) from about 4,500 individuals recruited in the French cohort D.E.S.I.R. (*Data from an Epidemiological Study on the Insulin Resistance syndrome*), we assess the feasibility of implementing the joint modelling approach in a real high-throughput genomic dataset. An alternative model approximating the joint model, called the Two-Step approach (TS), is also presented. Although the joint model shows more precise and less biased estimators than its alternative counterpart, the TS approach results in much reduced computational times, and could thus be used for testing millions of SNPs at the genome-wide scale.

## 1. Introduction

With the increased availability of longitudinal and survival data in large cohorts, joint models have emerged as an appropriate approach to account for both types of data, especially when dealing with informative/non-informative dropouts which commonly occur in such cohorts. Joint models have been studied and overviewed in the literature (Wulfsohn and Tsiatis, 1997; Tsiatis and Davidian, 2004; Chen et al., 2011; Elashoff et al., 2016), and their implementation has been proposed in different softwares and platforms (Diggle and Kenward, 1994; Sun et al., 2007; Elashoff et al., 2008; Proust-Lima et al., 2009; Rizopoulos, 2010; Rizopoulos and Ghosh, 2011). Main applications of the joint model approach are: (i) to efficiently model the survival process with a time-varying covariate, accounting for missing data and measurement error; and (ii) to account for informative dropouts in the longitudinal data. To model the two processes of a joint model, a linear mixed effects (LME) model and a Cox proportional hazards model (CoxPH) are classically used to, respectively, fit the longitudinal component and the survival component. Unlike the CoxPH model, in which the time-varying covariate is assumed to be exogenous, i.e., not modified by the occurrence of an event (Kalbfleisch and Prentice, 2002), the joint modelling framework allows to account for an endogenous time-varying covariate. An example of an endogenous covariate is the fasting blood plasma glucose which is irremediably modified due to glucose lowering medication, once T2D is diagnosed.

Two approaches can be used for estimation and inference of the model parameters: a “naive” two-step (TS) method or a joint likelihood method (JM). In the first method, the random effects of the trajectory are estimated by an LME model, then included as a time-varying covariate in a CoxPH model and estimated using the partial likelihood approach (Therneau and Grambsch, 2000). The second method is based on a joint likelihood of the two stochastic components (longitudinal and survival) estimated at the same time. Comparison of these two approaches showed that the latter offers more consistent and efficient estimators than the former (Albert and Shih, 2010a,b). But JM can be challenging to compute, especially when it comes to achieving convergence during the Expectation-Maximisation (EM) step. Moreover, depending on the number of time points and/or the sample size, the overall computational time can substantially increase.

In this paper, we conducted a comprehensive simulation study to compare these two approaches, JM and TS, when jointly modelling the longitudinal and the survival components, under the case of univariate variable for the longitudinal trait. Our main goal is to show that the JM approach, when compared to TS, increases statistical power to detect an effect on either or both the longitudinal and the survival processes, while resulting in bias reduction in parameter estimation. We also showed that, while highly demanding computation and convergence issues might arise during JM computation, TS offers a good alternative to JM in greatly reducing computational time, especially when applied at the genome-scale level.

We also investigated and decomposed the computational time required by R package “JM” (Rizopoulos, 2010, 2017), and by the TS approach which combines R packages “survival” (Therneau, 2017) and “nlme” (Pinheiro et al., 2017).

Finally, we applied both approaches to a real dataset, the D.E.S.I.R. cohort (*Data from the Epidemiological Study on the Insulin Resistance syndrome*), which included 5,212 individuals with extensive phenotypic measurements recorded at four 3-yearly intervals, spanning a 9-year follow up. Individuals were genotyped using the Illumina Metabochip DNA array of nearly 200,000 SNPs (Voight et al., 2012). Relying on the conventional cross-sectional genome-wide association study design, the D.E.S.I.R. cohort was instrumental in identifying novel loci associated with prevalent type 2 diabetes (T2D) and fasting plasma glucose (FPG) level in normoglycaemic individuals (Sladek et al., 2007; Bouatia-Naji et al., 2008; Rung et al., 2009). We specifically focus on time-to-onset of T2D, in order to identify novel loci or to confirm published ones, which could simultaneously be associated with higher risk of developing T2D and/or increased FPG, consequently SNPs are rather analysed one at a time than as clusters. Our results were compared to the genetic variants reported in the literature (Vaxillaire et al., 2014; Welter et al., 2014) and to the meta-analyses published by large consortia, such as DIAGRAM (Morris et al., 2012) and MAGIC (Dupuis et al., 2010) consortia.

## 2. Methods

### 2.1. Model formulations

#### 2.1.1. Joint likelihood model (JM)

The standard formulation of the joint model involves two components: a longitudinal component and a time-to-event component. Let *n* denote the sample size, and *Y*_*ij*_ the longitudinal measurements collected for each individual *i* at time points *t*_*ij*_, *i* = 1, ⋯ , *n*, *j* = 1, ⋯ , *m*_*i*_, where *m*_*i*_ is the number of measurements on individual *i*. The longitudinal component (i.e., measurements) typically consists of a (generalised) linear mixed effect (LME) model, whose within-subject correlation matrix is modelled using random-effect parameter vector $b_{i}=\left(\begin{array}{c} \theta_{0i}\\ \theta_{1i} \end{array}\right)$.

Under the joint likelihood framework implemented in “JM” (Rizopoulos, 2010, 2017), within the class of “shared parameter models” (Rizopoulos, 2012; Elashoff et al., 2016), we define

(1) $$\begin{array}{l} Y_{ij}=X_{ij}+\epsilon_{ij} \end{array}$$

where *Y*_*ij*_ is the observed value and *X*_*ij*_ is the true (unobserved) value of the longitudinal measurement at time *t*_*ij*_ for individual *i*. The quantity ϵ_*ij*_ is a random error term usually assumed to be normally distributed:

(2) $$\begin{array}{l} \epsilon_{ij}\sim N\left(0,\sigma^{2}\right) \end{array}$$

The quantity *X*_*ij*_ is typically called the trajectory function, and is usually specified as a linear or quadratic function of time *t*_*ij*_; for simplicity here, we assume linearity over time. We also define *Z*_*i*_, a vector denoting the genotype of individual *i*, and *W*_*i*_, a set of adjusting covariates:

(3) $$\begin{array}{l} Y_{ij}=X_{ij}+\epsilon_{ij}=\theta_{0i}+\theta_{1i}t_{ij}+\gamma Z_{i}+\delta W_{i}+\epsilon_{ij} \end{array}$$

Again, without any loss of generality, we omit the term δ*W*_*i*_ in the following. Random effects θ_0*i*_ (intercept) and θ_1*i*_ (slope) are assumed bivariate Normal: $\theta \sim N_{2}\left(\mu ,\Sigma \right)$, and independently distributed from ϵ_*ij*_. The coefficient γ assesses the genotypic (additive) effect of variable *Z*_*i*_ on the trajectory function. To account for varying slopes, an interaction term between *Z*_*i*_ and time *t*_*ij*_ could be added into the trajectory function; for simplicity, this term was not considered in this study.

The time-to-event (survival) component usually consists of a parametric (e.g., exponential or Weibull distribution) or semi-parametric (e.g., Cox proportional hazards) model. *T*_*i*_ denotes the event time for individual *i*, and *C*_*i*_ the right censoring time (end of the follow-up). Let Δ_*i*_ be the event indicator: Δ_*i*_ = 0, if *T*_*i*_ > *C*_*i*_, and Δ_*i*_ = 1, if *T*_*i*_ ≤ *C*_*i*_. Under the Cox proportional hazards model, variable *T*_*i*_ is specified using the following equation:

(4) $$\begin{array}{l} \lambda_{i}\left(t\right)=\lambda_{0}\left(t\right)\exp\left(\beta X_{i}\left(t\right)+\alpha Z_{i}+\eta W_{i}\right) \end{array}$$

where λ_*i*_(*t*) is the hazard function at time *t* for individual *i* and λ_0_(*t*) is the unspecified baseline hazard function, which we assume piecewise constant with two knots placed at intermediate time points in the follow-up. Coefficient α measures the effect of *Z*_*i*_ on the hazard function, while β measures the association between the trajectory function and the hazard function. In this formulation, we suppose that the subject-specific random effect parameters $b_{i}=\left(\begin{array}{c} \theta_{0i}^{*}\\ \theta_{1i}^{*} \end{array}\right)$ included in the trajectory *X*_*i*_(*t*) could modify the hazard function, which implies that β is the parameter linking the longitudinal and survival components.

#### 2.1.2. Two-step model (TS)

As an alternative to JM, and based on the work of Tsiatis et al. (1995), the two-step model estimates parameters of the joint model by first, estimating parameters of the trajectory function *X*_*i*_(*t*) in Equation (3), and second, by substituting this estimated trajectory, say $X_{i}^{*}\left(t\right)$, into Equation (4) before fitting the Cox survival model.

### 2.2. Simulations

Simulations were carried out to further examine the sensitivity of the JM estimations under several scenarios. Parameters were set based on values estimated (Table 1) from the strongest SNPs associated with T2D from the literature, i.e., rs17747324 in gene *TCF7L2* (T2D risk allele: C; α = 0.358; *p* = 8.5 × 10^−55^ (Morris et al., 2012); FPG increasing allele: C; γ = 0.025; *p* = 6.5 × 10^−08^ Dupuis et al., 2010).

**Table 1:** Parameters and numerical values used for sensitivity analysis and simulations, based on results from rs17747324 within gene *TCF7L2* in the French cohort D.E.S.I.R.

**Parameters**	**Values**
Number of participants (*n*)	4,352
Number of measures (*m*)	4
Diabetes incidence rate (*d*)	0.0384
Minor allele frequency (*f*)	0.244
Random effects (θ)	$\sim N_{2}\left([\begin{array}{c} 4.55\\ 0.0108 \end{array}],[\begin{array}{cc} 0.143 & -0.00109\\ -0.00109 & 6.8\times 10^{-04} \end{array}]\right)$
SNP effect on *Y*_*ij*_ (γ)	0.0229
SNP effect on *T*_*i*_ (α)	0.265
Association between *Y*_*ij*_ and *T*_*i*_ (β)	3.17
Error term (ϵ)	$\sim N\left(0,0.305^{2}\right)$

Longitudinal data were simulated according to Equation (3), while event times were generated from an exponential distribution for the CoxPH model (Austin, 2012), with *X*_*i*_(*t*) as a linear function.

(5) $$\begin{array}{l} \lambda_{0}\left(t\right)=\lambda \end{array}$$

(6) $$\begin{array}{l} H_{i}\left(T_{i}\right)=\int_{0}^{T_{i}}\lambda \exp\left(\beta X_{i}\left(t\right)+\alpha Z_{i}\right)dt \end{array}$$

(7) $$\begin{array}{l} F_{i}\left(T_{i}\right)=1-exp\left(-H_{i}\left(T_{i}\right)\right)=u \end{array}$$

(8) $$\begin{array}{l} u\sim U\left(0,1\right) \end{array}$$

(9) $$\begin{array}{l} T_{i}=\frac{1}{\beta \theta_{1i}}\log\left(1-\frac{\beta \theta_{1i}\times \log\left(1-u\right)}{\lambda \exp\left(\beta \theta_{0i}+\left(\beta \gamma +\alpha \right)Z_{i}\right)}\right) \end{array}$$

where λ was set to achieve the targeted incidence rate in the simulated dataset.

Datasets were simulated by varying the number of longitudinal measurements *m* ∈ {2; 3; 4; 5}, the number of individuals *n* ∈ {500; 1, 000; 2, 500; 5, 000; 10, 000}, the allele frequency *f* ∈ {0.05; 0.1; 0.25; 0.5} and the incidence rate *d* ∈ {0.025; 0.05; 0.1}, thereby leading to 240 different scenarios. Each scenario was simulated 500 times.

The Root-Mean Square Error (RMSE)

(10) $$\begin{array}{l} \text{RMSE}\left(\hat{\phi }\right)=\sqrt{E\left({\left(\hat{\phi }-\phi \right)}^{2}\right)} \end{array}$$

was used to assess precision of estimators ϕ = (β, γ, α), when testing the association between *Y*_*ij*_, and *T*_*i*_, the effect of *Z*_*i*_ on *Y*_*ij*_ and the effect of *Z*_*i*_ on *T*_*i*_, respectively. In addition, statistical power and type I error were also estimated. The computational burden of each approach (JM and TS) was also investigated as our goal is to implement these approaches at a genome-wide scale.

### 2.3. Computational times

Based on our simulations, we calculated approximate computational times for four sample sizes with parameters as listed in Table 1, using a UNIX system with Intel® Xeon® CPU E7- 4870 @ 2.40 GHz (80 such CPUs available computing in parallel). Table 2 shows computational times for one SNP, and for extrapolating the total computational time for 100,000 SNPs, which is the approximate number of SNPs on the Metabochip, after data cleaning and quality-control for common SNPs (minor allele frequency > 0.05).

**Table 2:** Approximate computational times using function “*system.time*” of R software.

	**Joint model**		**Two-step model**	
**Sample size**	**Mean (sd) per SNP in seconds**	**100 K SNPs in days**	**Mean (sd) per SNP in seconds**	**100 K SNPs in days**
500	51 (3.4)	59	0.71 (0.066)	0.82
2,500	100 (11)	120	3.1 (0.092)	3.6
5,000	180 (25)	210	6.3 (0.17)	7.3
10,000	340 (34)	400	9 (0.22)	10

*System time was computed ten times per sample size (number of individuals). Extrapolation is displayed for 100,000 SNPs*.

To investigate further computational time issues, we profiled the execution of the main function “*jointmodel*” from the R package “JM,” which implements the joint likelihood modelling approach as described in this paper. In the “JM” package, the linear mixed effect sub-model is handled by the function “*lme*” from the “nlme” package. One may argue that using a faster approach, e.g., as implemented in the R package “lme4”, might decrease the computational time.

### 2.4. Real data

**Figure 1:**
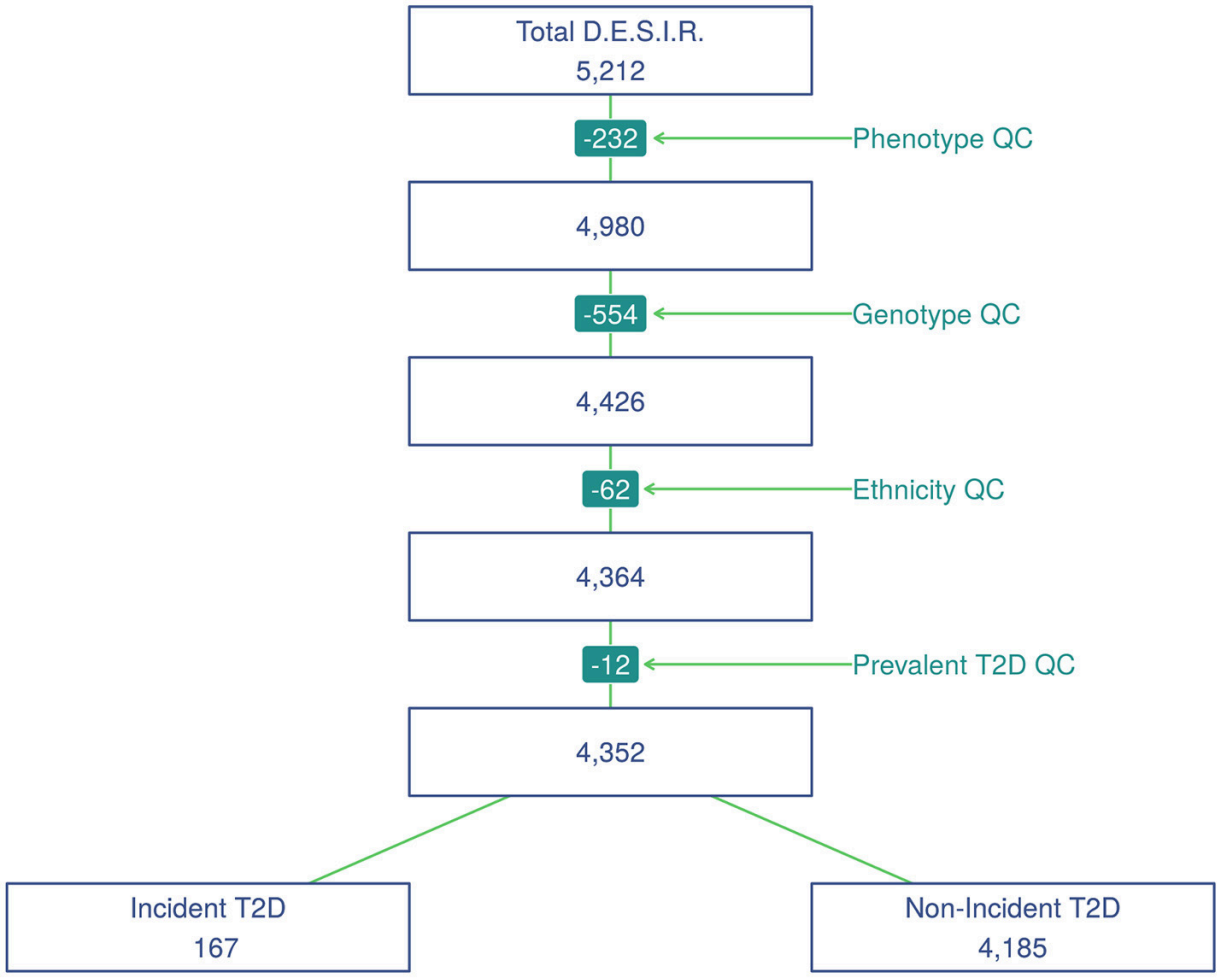
Study flowchart of participants from the French cohort D.E.S.I.R.

SNP genotyping was performed with Metabochip DNA arrays (Voight et al., 2012) using Illumina HiScan technology and GenomeStudio software (Illumina, San Diego, USA) in 5,212 individuals from the French cohort D.E.S.I.R. (Balkau, 1996). These participants have been followed for 9 years, and extensive phenotypic data has been recorded at four different 3-yearly time interval during that follow-up. All participants signed informed consent, and the protocol was approved by the ethics committee of Kremlin Bicetre Hospital, Paris. Quality control was performed using PLINK 1.90 beta version (Chang et al., 2015; Purcell and Chang, 2015). SNPs with call rate of at least 95%, with no significant deviation from Hardy-Weinberg equilibrium at *p* > 1 × 10^−5^, and with minor allele frequency (MAF) over 5% were kept for analysis, resulting in 101,305 SNPs. Due to missing phenotypes which did not allow to confirm T2D status, 232 individuals were removed. An additional 554 individuals were excluded due to individual call rate lower than 95%, leaving 4,426 individuals for analysis after these quality control steps (Figure 1).

Principal component analysis was performed using a combined dataset comprised of the 4,426 D.E.S.I.R. participants, along with participants from the publicly available 1,000 Genomes database (The 1000 Genomes Project Consortium, 2015). SNPs retained for analysis were restricted to those common to both sample sets. The first two components were sufficient to discriminate ethnic origin, which led to exclusion of 62 non Caucasians. A further 12 prevalent cases of T2D at baseline were also removed. As a result, the final dataset included 4,352 individuals, of whom 167 were diagnosed as T2D incident cases. Type 2 diabetes was defined using one of the following criteria: use of glucose lowering medication, and/or fasting plasma glucose [*FPG*] ≥ 7 mmol/L, and/or glycaeted haemoglobin A1c [*HbA*1*c*] ≥ 6.5% (48 mmol/mol).

**Figure 2:**
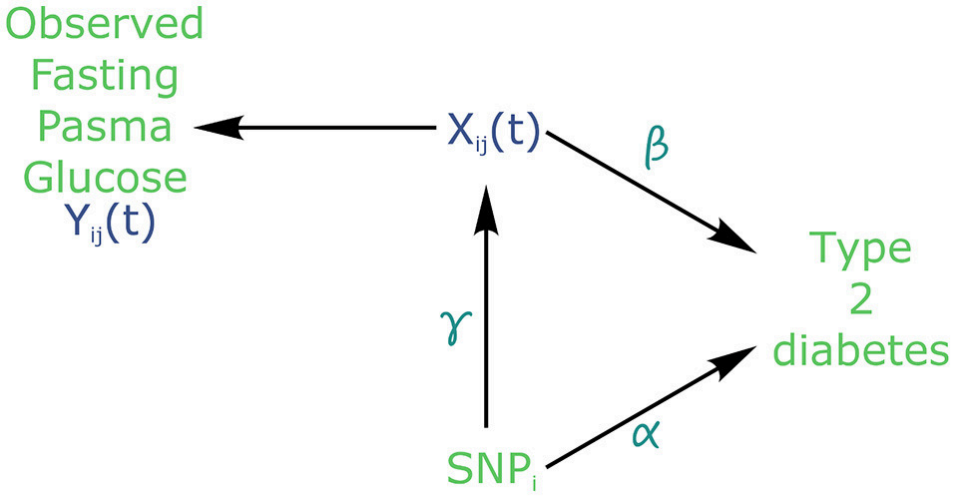
Causal diagram for joint modelling applied to fasting plasma glucose (FPG) and type 2 diabetes (T2D) (adapted from Ibrahim et al., 2010). SNP: Single Nucleotide Polymorphism. *X*_*ij*_ is the true (unobserved) FPG trajectory. β measures the association between FPG trajectory and T2D incidence. exp(α) measures the hazard ratio for T2D. γ measures the association between a *SNP*_*i*_ and FPG trajectory.

Using the joint modelling approach implemented in the package “JM” (Rizopoulos, 2010, 2017) within the R software version 3.4.2 (R Core Team, 2017), all 101,305 SNPs were tested for joint association with FPG and T2D. Following the above joint modelling formulation, *Y*_*ij*_ denotes the observed values of FPG, *Z*_*i*_ represents the genotype of individual *i* at each SNP, with *W*_*i*_ being covariates such as age, sex and BMI (Figure 2). Finally, *T*_*i*_ gives the time at which an individual is diagnosed with T2D.

In the joint modelling framework, the trajectory of FPG could be viewed as a dropout process, because all FPG values are flagged as missing after T2D diagnosis. In effect, individuals receiving a diabetes diagnostic are immediately placed under treatment to lower and regulate their blood glucose level. Therefore, FPG must be considered as an endogenous covariate, because the dropout process is not independent from the measured glucose values prior to T2D diagnosis.

## 3. Results

### 3.1. Comparison of estimation accuracy

Due to the complexity of the estimating algorithm within JM, convergence could not be obtained (4.53% of convergence issues on average per scenario, with a standard deviation of 5.81%) for the whole set of 500 simulations (i.e., algorithm “piecewise-PH-aGH” for a time-dependent relative risk model with a piecewise constant baseline risk function, using the adaptive Gauss-Hermite quadrature rule to approximate integrals within the Expectation-Maximisation (EM) step; Rizopoulos, 2010, 2017).

**Figure 3:**
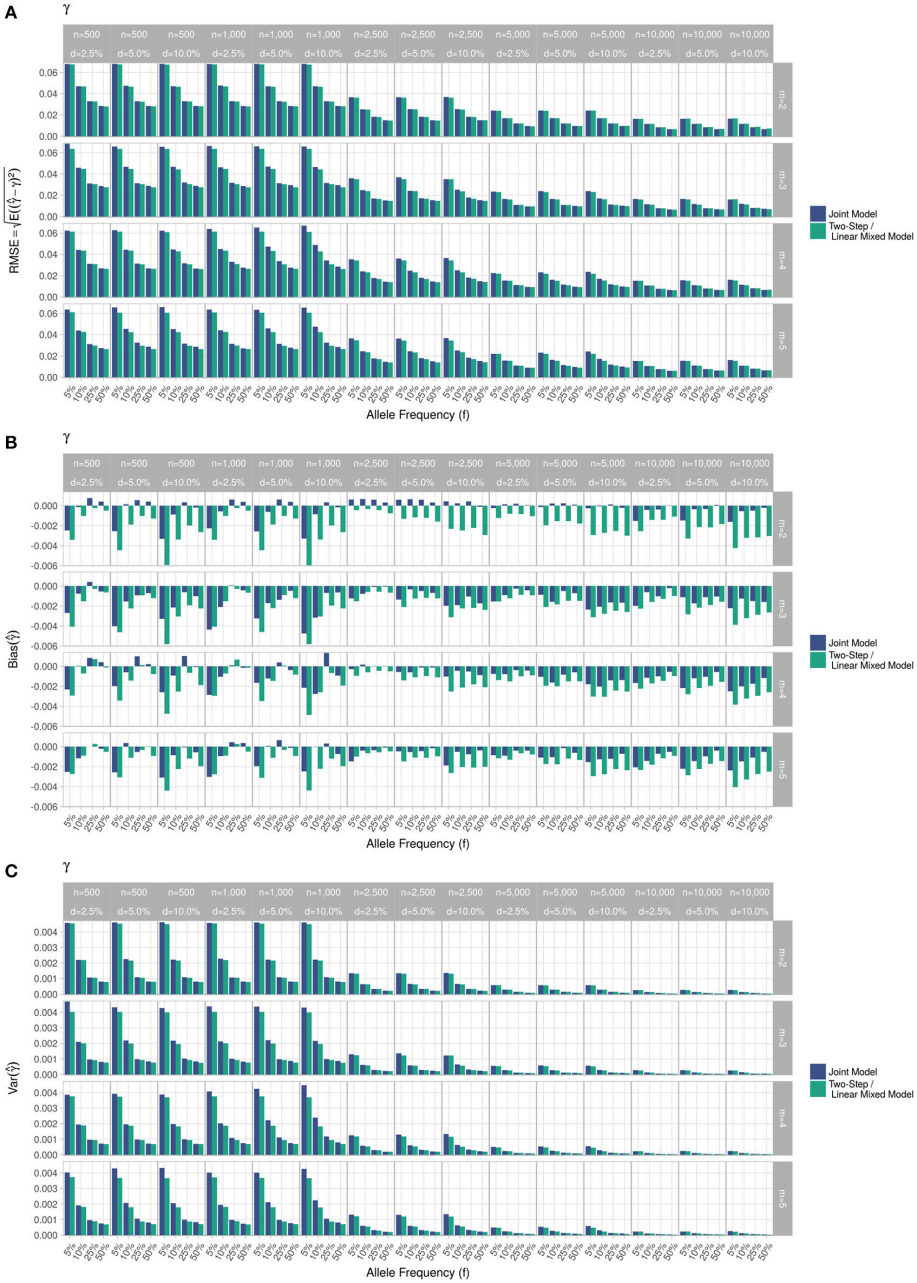
Simulation study for accuracy of estimator $\hat{\gamma }$ provided by the joint model (“JM” package) and by the two-step linear mixed effect model (“nlme” package). **(A)** Displays RMSE, **(B)** Displays bias and **(C)** Displays variance. *m*, number of measures; *n*, number of individuals; *d*, diabetes incidence rate.

**Figure 4:**
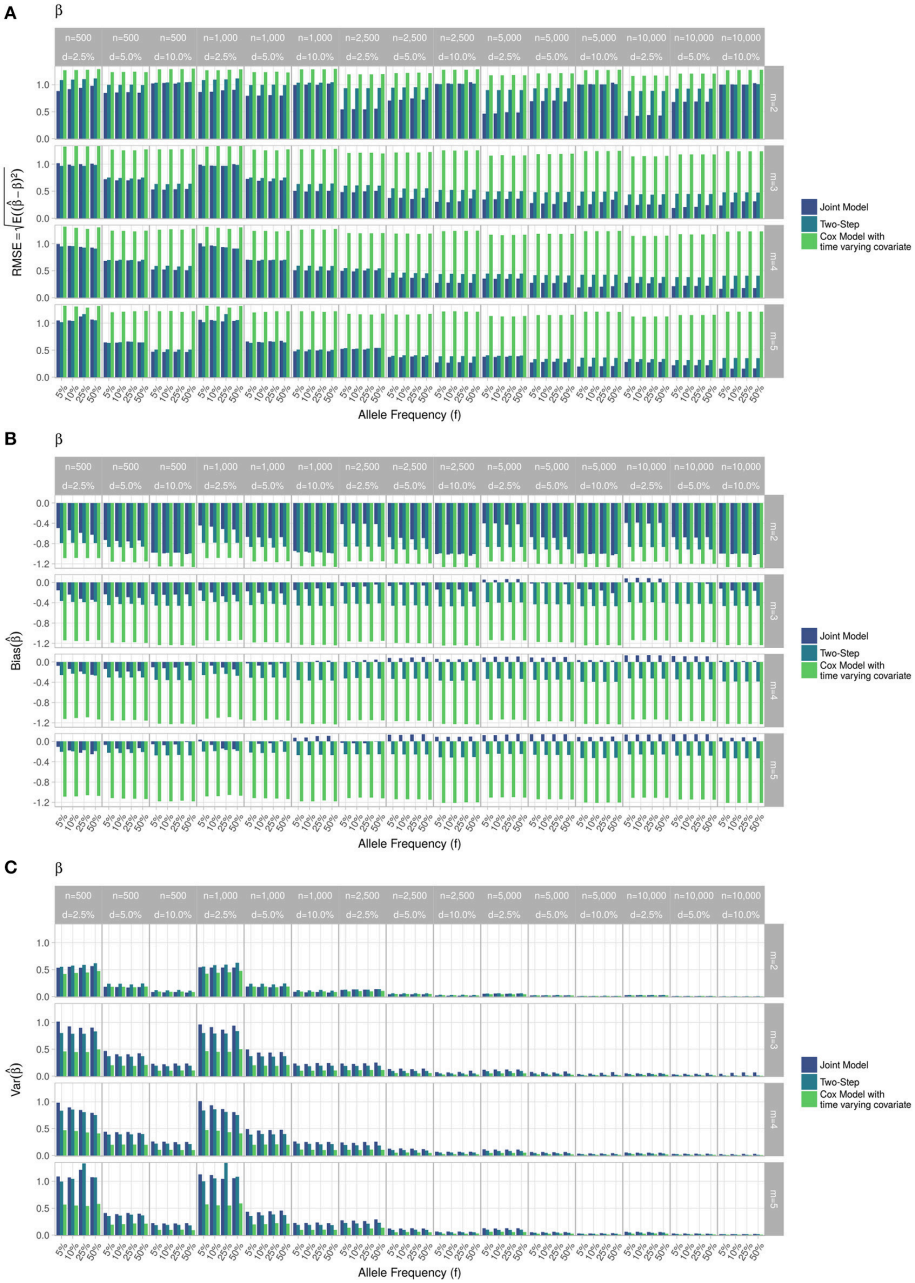
Simulation study for accuracy of estimator $\hat{\beta }$ provided by the joint model (“JM” package), by the two-step linear mixed effect model (“nlme” package) and by the Cox model with time-varying covariate. **(A)** Displays RMSE, **(B)** Displays bias and **(C)** Displays variance. *m*, number of measures; *n*, number of individuals; *d*, diabetes incidence rate.

**Figure 5:**
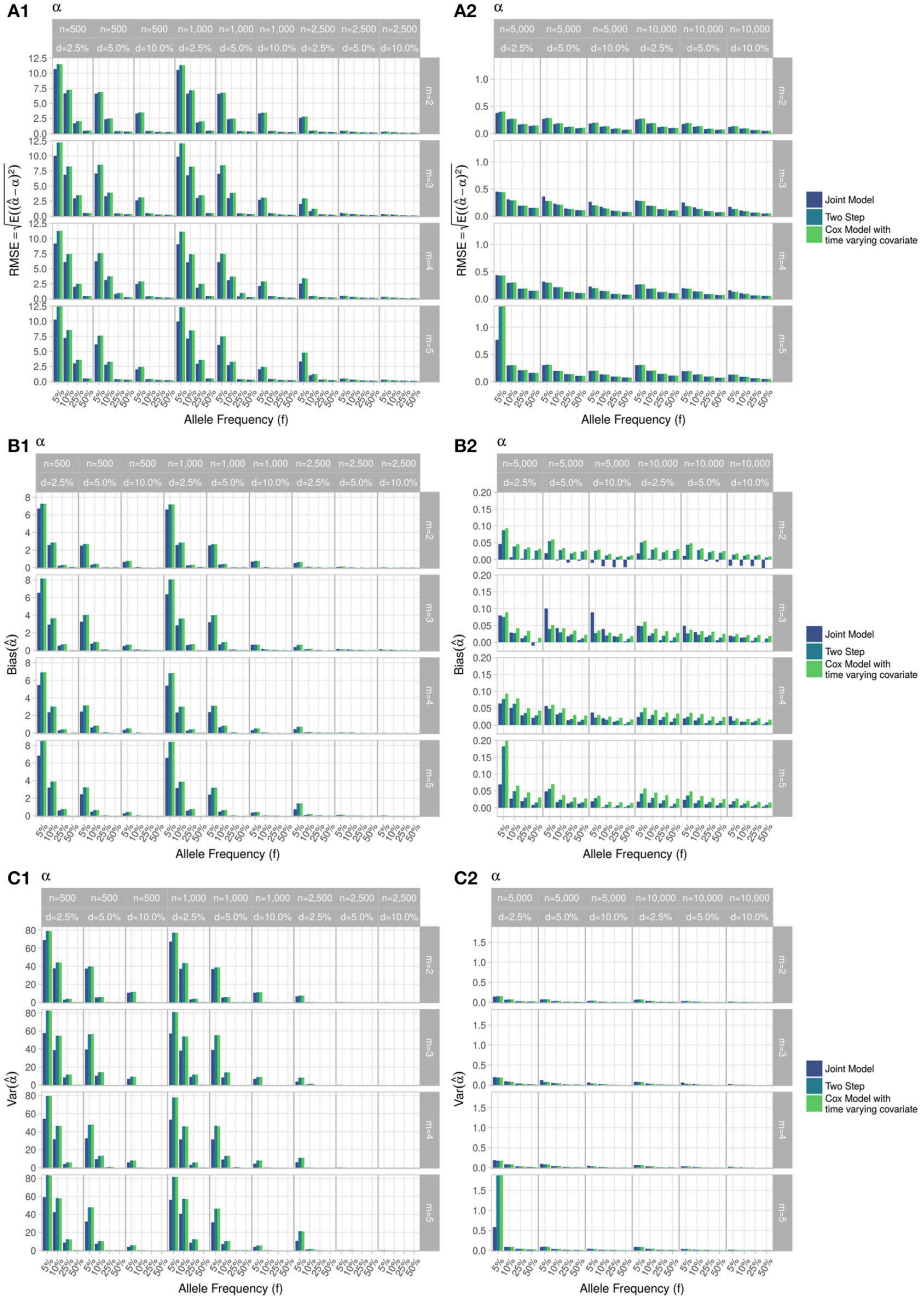
Simulation study for accuracy of estimator $\hat{\alpha }$ provided by the joint model (“JM” package), by the two-step linear mixed effect model (“nlme” package) and by the Cox model with time-varying covariate. *m*, number of measures; *n*, number of individuals; *d*, diabetes incidence rate. **(A)** Displays RMSE, **(B)** Displays bias and **(C)** Displays variance. (1) Displays results for *n* = 500 to *n* = 2, 500. (2) Displays results for *n* = 5, 000 to *n* = 10, 000.

RMSE for parameter γ (Figure 3) showed similar performance for JM and TS. RMSE for parameter β (Figure 4) and for parameter α (Figure 5) were smaller within the joint modelling framework (either JM or TS) than in the more classical CoxPH model with time-varying fasting plasma glucose. While the RMSE for β remains the same in the CoxPH model across all scenarios, under JM or TS it decreased whenever the sample size, the incidence rate or the allele frequency increases. Differences in RMSE for parameter α were smaller than for parameter β. Both TS and CoxPH with time-dependent covariate performed similarly, probably because partial likelihood inferences were used in these two approaches. JM estimations, for β and γ, were less biased in almost all scenarios when the sample size was >2,500. The larger bias observed within the extended CoxPH model, especially for β, could be explained by the mischaracterisation of the measurement error in the longitudinal trait.

Overall, our simulations showed that JM is less biased than when separate approaches are used to model the effect of *Z*_*i*_ on the longitudinal process *Y*_*i*_, and on the time-to-event *T*_*i*_. While separate approaches performed well for parameters γ and α, the bias for parameter β was the highest across all scenarios.

### 3.2. Computational time

Computational times are reported in Table 2. The time required to complete JM or TS algorithms increased linearly with sample size in our simulations. However, these times are very optimistic since our simulations did not include any covariate or more complex random parameters. The main issue appears within the “*jointmodel*” function which took over 95% of the global computation time. After examination of the call tree diagram, we observe that the more time-consuming task within the “*jointmodel*” function happens during the optimisation of the EM algorithm (described in Rizopoulos, 2012, Appendix B), despite the use of a calculation trick (i.e., adaptive Gauss-Hermite quadrature for numerical integration).

### 3.3. Application in real data

**Figure 6:**
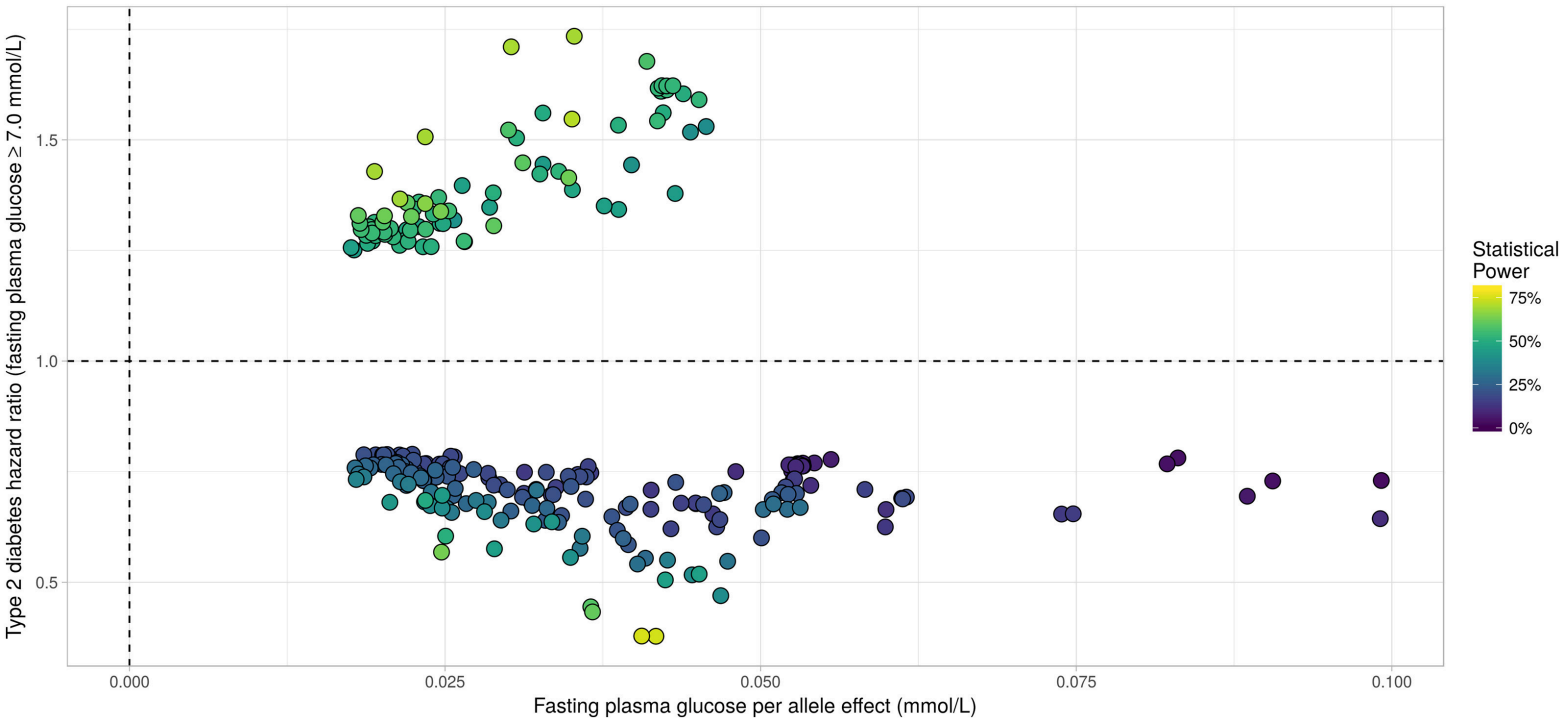
Results from statistical analysis using “JM” (Rizopoulos, 2010, 2017), using fasting plasma glucose increasing allele as effect allele. Estimated effects of γ are displayed on the x-axis, with corresponding estimated hazard ratio exp(α) on the y-axis. Statistical power reported is the theoretical (retrospective) power to detect a genetic joint effect βγ+α based on estimated model parameters (Chen et al., 2011).

Applying the R package “JM” to our D.E.S.I.R. cleaned dataset, 265 SNPs (Figure 6) were associated (with *p* < 0.05) with FPG and T2D events through their respective parameters γ and α. Amongst these 265 SNPs (163 unique genes), we identified 17 genes (Table 3) which had already been reported to be associated with FPG and/or T2D risk. Parameter β was highly significant (below the genome-wide threshold of 5 × 10^−8^) for all these SNPs, which was expected considering that β estimates the association between FPG trajectory and T2D risk.

**Table 3:** List of loci found to be associated within the joint modelling framework with both FPG and T2D risk, previously shown as associated with FPG and/or T2D risk in the NHGRI GWAS Catalogue (Welter et al., 2014).

**SNP (gene)**	**α (*p*-value)**	**γ (*p*-value)**	**β (*p*-value)**	**Power (*βγ* + α)%**	**Risk allele frequency**
rs6945660_G (*ETV1*)	0.550 (3.7 × 10^−02^)	0.035 (2.5 × 10^−02^)	3.480 (9.6 × 10^−45^)	69.7	0.91
rs1942873_C (*MC4R*)	0.410 (1.3 × 10^−02^)	0.023 (3.7 × 10^−02^)	3.140 (1.9 × 10^−41^)	69.6	0.81
rs55899248_G (*TCF7L2*)	0.292 (2.7 × 10^−02^)	0.025 (1.7 × 10^−02^)	3.490 (1.7 × 10^−44^)	55.3	0.24
rs17301514_A (*ST6GAL1*)	−0.657 (4.4 × 10^−03^)	0.045 (3.4 × 10^−03^)	3.650 (2.9 × 10^−45^)	45.8	0.09
rs833425_C (*PTPRD*)	0.321 (5.0 × 10^−02^)	0.043 (4.2 × 10^−03^)	3.510 (1.3 × 10^−43^)	44.2	0.10
rs7072870_A (*C10orf35*)	−0.404 (7.5 × 10^−03^)	0.025 (2.2 × 10^−02^)	3.580 (1.7 × 10^−45^)	39.6	0.22
rs61871514_A (*KCNQ1*)	0.425 (4.7 × 10^−02^)	0.046 (2.0 × 10^−02^)	3.180 (8.5 × 10^−42^)	39.4	0.06
rs9883865_A (*ADAMTS9*)	−0.598 (7.5 × 10^−04^)	0.043 (1.2 × 10^−02^)	3.200 (5.9 × 10^−42^)	34.9	0.92
rs114508985_C (*HLA*)	−0.294 (2.1 × 10^−02^)	0.021 (3.0 × 10^−02^)	3.220 (8.2 × 10^−43^)	27.1	0.31
rs10814856_T (*GLIS3*)	−0.265 (4.0 × 10^−02^)	0.025 (1.5 × 10^−02^)	3.200 (1.5 × 10^−42^)	18.5	0.73
rs73025532_C (*SLC22A1*)	−0.377 (4.8 × 10^−02^)	0.032 (3.6 × 10^−02^)	3.580 (1.3 × 10^−45^)	17.3	0.90
rs11769484_C (*JAZF1*)	−0.254 (4.8 × 10^−02^)	0.022 (3.6 × 10^−02^)	3.210 (2.1 × 10^−42^)	16.9	0.77
rs6450176_G (*ARL15*)	−0.291 (1.8 × 10^−02^)	0.036 (3.0 × 10^−04^)	3.540 (2.2 × 10^−45^)	15.2	0.73
rs4712580_C (*CDKAL1*)	−0.289 (4.2 × 10^−02^)	0.031 (7.4 × 10^−03^)	3.570 (1.2 × 10^−45^)	14.0	0.82
rs10830963_G (*MTNR1B*)	−0.440 (9.4 × 10^−04^)	0.099 (1.3 × 10^−23^)	3.250 (3.6 × 10^−42^)	10.2	0.29
rs853787_T (*ABCB11*)	−0.247 (4.3 × 10^−02^)	0.083 (9.3 × 10^−19^)	3.210 (1.7 × 10^−42^)	03.3	0.65
rs560887_C (*G6PC2*)	−0.315 (1.2 × 10^−02^)	0.099 (9.6 × 10^−25^)	3.210 (1.3 × 10^−42^)	02.6	0.70

**Figure 7:**
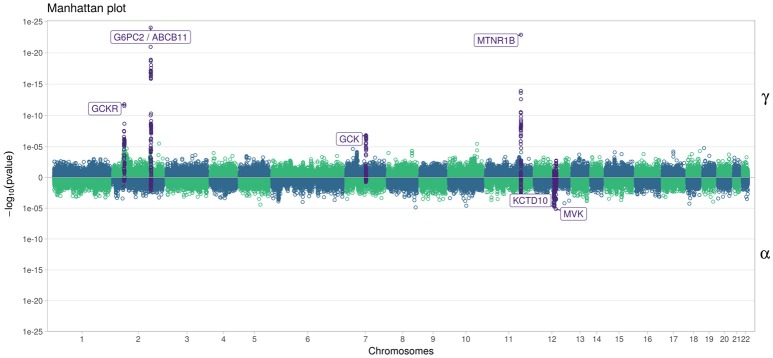
Manhattan plot for estimated effects of γ and α using “JM” (Rizopoulos, 2010, 2017). Results are presented for the cleaned set of 101,305 SNPs.

In Figure 7, we specifically focused on parameters γ and α. After Bonferroni correction (nominal *p*-value ≃ 5 × 10^−7^), no genetic variants showed a highly significant association with both parameters γ and α simultaneously; only SNPs in the following genes (or within a 100 kb window) remained significant when testing for γ: *G6PC2*/*ABCB11, GCK*/*YKT6, GCKR*, and *MTNR1B*, with per-allele increasing effect varying on FPG from 0.100 to 0.047 mmol/L (data not shown). Zooming in on simultaneous associations with the longitudinal and survival processes revealed well known genes, such as *TCF7L2*, which has been shown in many meta-analyses to be associated with elevated FPG and an increased risk of T2D (Table 4). *MTNR1B* was also found to be associated (34 SNPs within 30 kb) with estimated $\hat{\alpha }=-0.44$ (*p* = 9.37 × 10^−04^) and $\hat{\gamma }=0.099$ (*p* = 1.33 × 10^−23^) for SNP rs10830963, the SNP usually reported in the literature.

**Table 4:** Effect sizes on FPG and T2D risk estimated using JM.

	α **(*****p*****-value)**		γ **(*****p*****-value)**		**β (*p*-value)**
**SNP (gene)**	**JM (D.E.S.I.R.)**	**DIAGRAM**	**JM (D.E.S.I.R.)**	**MAGIC**	**JM (D.E.S.I.R.)**
rs10830963_G (*MTNR1B*)	−0.44 (9.4 × 10^−04^)	0.104 (7.3 × 10^−07^)	0.0991 (1.3 × 10^−23^)	0.079 (1.3 × 10^−68^)	3.25 (3.6 × 10^−42^)
rs17747324_C (*TCF7L2*)	0.265 (4.1 × 10^−02^)	0.358 (8.5 × 10^−55^)	0.0229 (3.0 × 10^−02^)	0.025 (6.5 × 10^−08^)	3.17 (8.9 × 10^−42^)

*Comparison is shown with effect sizes as reported by consortia meta-analyses in genes MTNR1B and TCF7L2*.

**Figure 8:**
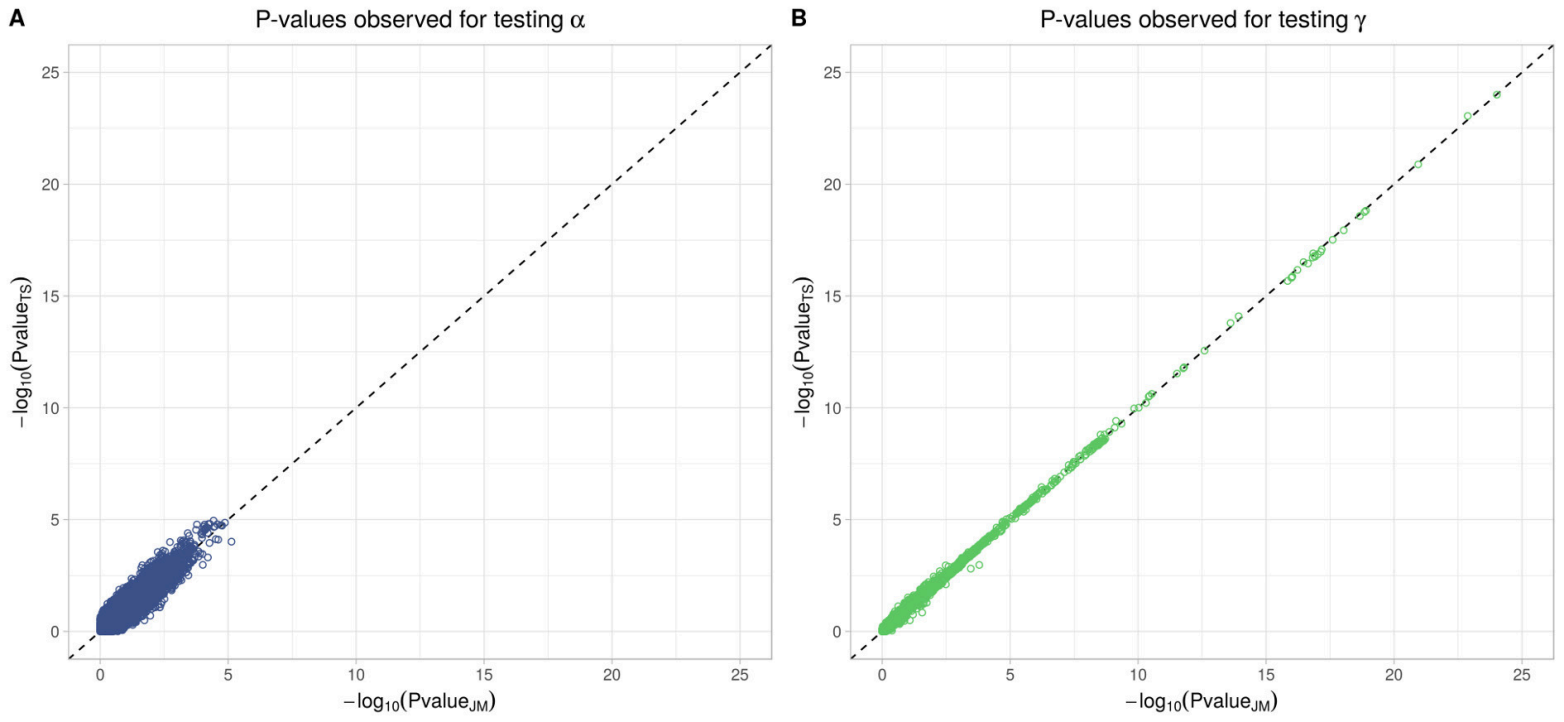
Testing for α (SNP effect on onset of T2D) and γ (SNP effect on the trajectory of FPG) using Two-Step approach compared to Joint Model approach. On the x-axis, −log_10_(*p*-value) from the Joint Model and on the y-axis the corresponding −log_10_(*p*-value) from the approximate Two-Step approach. **(A)** Displays −log_10_(*p*-value) for testing α parameter from Equation (4). **(B)** Displays −log_10_(*p*-value) for testing γ parameter from Equation (3).

To better compare JM and TS, we repeated the analysis on the whole dataset using TS. As shown in Figure 8, *p*-values differ, especially when testing parameter α; however for tests on parameter γ, approximations were quite close to the *p*-values provided via the joint likelihood framework.

## 4. Discussion

With the ever-increasing availability of genomic data generated by genotyping arrays and next generation sequencing, the need to develop and implement efficient models is important to ensure that statistical analysis will be achieved in a reasonable timeframe. In this paper, we proposed a comparison of two approaches, namely the joint model (JM) and the two-step model (TS), to estimate parameters accounting simultaneously for a genetic effect on both longitudinal and survival processes without discarding missing values or dropouts commonly generated by the longitudinal measurement process. In our real data application, FPG serves as the longitudinal process, whereas T2D diagnosis generates survival times of interest, both stochastic phenomenon being linked by the fact that a fixed threshold for FPG defines T2D onset (currently, [*FPG*] ≥ 7 mmol/L), along with glucose lowering medication use. Through simulations over different scenarios, we showed that joint models are less biased than classical separate approaches. Hopefully, joint models could provide more insight regarding the event of interest, and could assess the potential impact of a genetic marker on incident T2D better than simpler models.

By looking at statistical measures of accuracy such as RMSE for our model estimators, and by estimating the computational time required by the available R implementations of joint models, our study showed that the use of an approximate method at a genome-wide scale, such as TS, might represent a good compromise between accuracy and computational time. TS could be used to overcome the computational burden of current joint likelihood methods by exploiting available R packages performing the two steps, LME and CoxPH, and could help filter out SNPs with low or undetectable associations during a first preliminary scan. However, depending on the parameters of the dataset (sample size, incidence rate, number of measures), a joint likelihood method always has to be preferred over TS when one wants to obtain accurate estimation of parameters γ and α, which describe the SNP effect on the trajectory of FPG and on the time-to-onset of T2D, resp. Although we computed the theoretical statistical power to detect a joint genetic effect βγ+α (Chen et al., 2011), we did not test this effect at the genome-wide scale due to its computational burden. In this paper, we used the closed-form expression from Chen et al. (2011) to evaluate retrospectively the probability to obtain the same significant results in a similarly designed study to D.E.S.I.R. This closed-form expression could be use to design a new study (e.g., compute the number of samples) which aims at identifying a joint effect. The joint SNP effect can be tested using a likelihood ratio test comparing the full joint model, i.e., with a SNP effect included in both sub-models, to the joint model without a SNP effect in the survival sub-model, as implemented in the package “JM” (Rizopoulos, 2010, 2017).

To fully characterise JM approach, further study needs to be performed, such as missing values distribution according to the usual hypotheses (i.e., Missing At Random, Missing Completely At Random and Missing Not At Random). In this paper, we did not study in our simulations the rates of change effect of the SNPs (i.e., interaction term *SNP* × *TIME*) which might also be of interest in the study of a disease such as T2D.

Finally, we would like to reemphasize that using parallel and grid computing approaches will help reduce the global computational time when applied at a genome-wide scale (i.e., with millions of SNPs).

In our real data application, rs17747324 showed consistent results with the DIAGRAM and MAGIC (for FPG) consortia for both α and γ (Table 4), but rs10830963 showed an opposite effect on T2D risk compared to the effect reported in the DIAGRAM consortium ($\hat{\alpha }=0.104$, *p* = 7.3 × 10^−07^). Results observed for *MTNR1B* (rs10830963) in the French cohort D.E.S.I.R., albeit inconsistent with previous studies, may uncover some interesting peculiarities pertaining to T2D incident cases in this population. In the literature, SNPs in *MTNR1B* were reported as being associated with higher FPG and T2D risk, but meta-analyses were performed on populations with different genetic backgrounds, and the two traits have never been jointly co-analysed. However, we realize that *MTNR1B* associations identified in our study need to be confirmed and replicated in other cohorts, as they might be cohort-specific. Finally, a major limitation in our study comes from the low number of incident T2D cases in the D.E.S.I.R. cohort (167 incident T2D cases in 4,352 individuals followed over 9 years), resulting in (retrospective) power, no higher than 70%, as shown in Figure 6 and Table 3.

## Data availability

The datasets for this manuscript are not publicly available due to consideration of intellectual property, ongoing active collaborations and to continuing analyses by the study investigators.

Requests to access the datasets should be directed to PF (p.froguel@imperial.ac.uk).

## Author contributions

GR and MC: contributed to the study conception and design; BB, PF, and RR: made the genomic and phenotypic data available; MC: conducted the data and statistical analyses; GR and MC: interpreted the data and results; MC: drafted the first version of the manuscript; BB, GR, PF, and RR: edited and provided critical revisions to the manuscript. All authors contributed to manuscript revision, read and approved the submitted version.

### Conflict of interest statement

The authors declare that the research was conducted in the absence of any commercial or financial relationships that could be construed as a potential conflict of interest.
